# Platelet-Rich Plasma and Skeletal Muscle Healing: A Molecular Analysis of the Early Phases of the Regeneration Process in an Experimental Animal Model

**DOI:** 10.1371/journal.pone.0102993

**Published:** 2014-07-23

**Authors:** Ivan Dimauro, Loredana Grasso, Simona Fittipaldi, Cristina Fantini, Neri Mercatelli, Silvia Racca, Stefano Geuna, Alessia Di Gianfrancesco, Daniela Caporossi, Fabio Pigozzi, Paolo Borrione

**Affiliations:** 1 Unit of Biology, Genetics and Biochemistry, Department of Movement, Human and Health Sciences, University of Rome “Foro Italico”, Rome, Italy; 2 Unit of Internal Medicine, Department of Movement, Human and Health Sciences, University of Rome “Foro Italico”, Rome, Italy; 3 Department of Clinical and Biological Sciences, University of Turin, Turin, Italy; Ohio State University, United States of America

## Abstract

Platelet-rich plasma (PRP) has received increasing interest in applied medicine, being widely used in clinical practice with the aim of stimulating tissue healing. Despite the reported clinical success, there is still a lack of knowledge when considering the biological mechanisms at the base of the activity of PRP during the process of muscle healing. The aim of the present study was to verify whether the local delivery of PRP modulates specific molecular events involved in the early stages of the muscle regeneration process. The right *flexor sublimis* muscle of anesthetized Wistar rats was mechanically injured and either treated with PRP or received no treatment. At day 2 and 5 after surgery, the animals were sacrificed and the muscle samples evaluated at molecular levels. PRP treatment increased significantly the mRNA level of the pro-inflammatory cytokines IL-1β, and TGF-β1. This phenomenon induced an increased expression at mRNA and/or protein levels of several myogenic regulatory factors such as MyoD1, Myf5 and Pax7, as well as the muscular isoform of insulin-like growth factor1 (IGF-1Eb). No effect was detected with respect to VEGF-A expression. In addition, PRP application modulated the expression of miR-133a together with its known target serum response factor (SRF); increased the phosphorylation of αB-cristallin, with a significant improvement in several apoptotic parameters (NF-κB-p65 and caspase 3), indexes of augmented cell survival. The results of the present study indicates that the effect of PRP in skeletal muscle injury repair is due both to the modulation of the molecular mediators of the inflammatory and myogenic pathways, and to the control of secondary pathways such as those regulated by myomiRNAs and heat shock proteins, which contribute to proper and effective tissue regeneration.

## Introduction

Musculoskeletal injures are the most common cause of severe long-term pain and physical disability, affecting hundreds of millions of people around the world and accounting for the majority of all sport-related injured [1]. In addition to health care expenditures, the social cost of these injuries includes lost job wages and production. In competitive or professional athletes, this loss may have extreme consequences.

During the last decade, the rapidly increasing understanding of the contribution of growth factors (GFs) in the healing of injured tissue has given rise to a great interest in the use of autologous platelet-rich plasma (PRP) as a new therapeutic tool in the field of dentistry, dermatology, plastic and maxillofacial surgery, acute trauma, chronic tendinopathies, cosmetic surgery, veterinary medicine, and in muscle strain injuries [2]–[8].

Autologous PRP is obtained from the separation of whole blood into plasma and red cell constituents, with the subsequent concentration of platelets into a small volume of plasma. Separation is frequently achieved by varying degrees of centrifugation but may equally occur via cell separator apparatus [9]. PRP is an example of autologous biomaterial product applied and studied since the 1980s [10]–[12].

The rationale for the widespread use of PRP resides in the fact that it is a simple, efficient, and minimally invasive method of obtaining a natural concentration of autologous GFs, which may modulate and regulate the tissue-healing process at a cellular level [13]. The rationale is that by the delivery of various GFs and cytokines from the α–granules contained in platelets, PRP enhances the recruitment, proliferation, and differentiation of cells involved in tissue regeneration.

Although there are several basic science studies, animal studies, and small case reports regarding PRP-related products, there are only a few controlled, clinical studies providing a high level of medical evidence when considering the potential benefits of PRP [2]–[8]. The number of participants in studies is typically small and the majority of studies are underpowered. Moreover, the studies examining the PRP effect have not used standardized techniques and the majority are anecdotal studies based on small case series. This is particularly evident when considering the treatment of musculoskeletal injuries, a field in which some interesting promising findings have been obtained, but most results are still preliminary and controversial.

Given the importance of an accelerated muscle regeneration, especially within the initial week of the muscle repair process where the inflammation and the regeneration phase take place, it is clear that further studies are required in order to recommend or discourage the adoption of this approach in routine clinical practice. Moreover, the biological mechanisms for the improved muscle recovery resulting from the use of PRP after injury need to be elucidated.

In the present study, we used our established and reproducible animal model to test the effects of autologous PRP on the molecular processes that characterize the early stages of muscle regeneration. On the basis of previous results [7], [14], we hypothesized that the local delivery of PRP into injured skeletal muscle accelerates specific processes such as the control of inflammatory reaction as well as myogenesis.

Since myoblast proliferation following myotrauma is orchestrated by multiple factors including growth factors, transcription factors, and miRNAs relating to myogenesis *in vivo*, in this study we analyzed at 2- and 5-day post-injury the multi-directionally effects of PRP on the expression of several cytokines (TNFα, IL-6, IL-10, IL-1β, and TGF-1β), myogenic response factors (MRFs) (MyoD1, Myf5, Pax7, Myogenin, and Mrf4), GFs (VEGF-A and IGF-1Eb), as well as myo-miRNAs (miR-1, miR-133a, and miR-206), stress-response proteins (Hsp70, Hsp27 and αB-crystallin) and apoptotic markers (NF-κB-p65, Bcl-2, Bax, and caspase 3).

## Materials and Methods

### Ethics Statement

All procedures were supervised by a veterinarian and approved by the Committee on the Ethics of Animal Experiments of the University of Turin in accordance with the European Communities Council Directive of 24 November 1986 (86/609/EEC).

All surgery was performed under sodium pentobarbital anesthesia, and all efforts were made to minimize animal suffering.

### Animals and surgery

Wistar male adult rats (n = 50), weighing approximately 250g each, were used. All animals were kept in a cage in a room with controlled temperature and humidity, with a light/dark cycle of 12/12 hours, and allowed food and water *ad libitum*. Animals were operated under general anesthesia by intramuscular injection of tiletamine + zolazepam (Zoletil) 3 mg/kg. The surgical procedures were performed with the aid of a surgical microscope (Zeiss OPMI7, Jena, Germany). A longitudinal incision was performed on the right anterior limb from the elbow region to the wrist in order to access the *flexor sublimis* muscles of the upper joint of the digits. The muscle was then injured transversely and medially using a scalpel. The wedge-shaped lesion was 3 mm long, 2 mm wide and 3 mm deep (see panel D in Figure S1).

To evaluate a possible effect of PRP on muscle regeneration process, an injury was made to the right flexor muscles of 20 rats and immediately treated with PRP (PRP group), 20 rats were subjected to the same muscle injury and left untreated (NO-PRP group), 10 rats, used as controls, were left uninjured (Ctrl group). After recovery from anesthesia, animals returned free in their cages. They were sacrificed at day 2 and 5 post-surgery and all muscle samples were immediately either frozen in liquid nitrogen and stored at −80°C until analyses were performed (protein and RNA analysis) or processed for immunohistochemistry.

### Blood collection and preparation of platelet-rich plasma

Blood was collected by intracardiac puncture of the anesthetized rats immediately before the surgical injury to the muscle (see panel A in Figure S1). Briefly, a needle (21G) was inserted at the base of the sternum at a 20° angle just lateral of the midline. The intracardiac blood (3–3.5 mL) was slowly withdrawn into a syringe containing 1 mL of 3.8% sodium citrate. Blood was then transferred into sterile tubes containing sodium citrate and underwent a first centrifugation at 220 g for 15 minutes (see panel B in Figure S1). To objectively determine the number of platelets and investigate the presence of other blood cells, a complete blood count was performed using a cell counter ADVIA 2021 (Bayer, Leverkusen, Germany) on a small amount of the plasma layer obtained after the first centrifugation (the white blood cell content was always very low: (0.01 ± 0.082)×10^3^/mm^3^, P-PRP subtype [9]. A second centrifugation at 1,270 *g* for 5 minutes allowed the platelets to fall to the bottom of the tube. The pellet was re-suspended in 100 µL of plasma (baseline range: 500–1300 ×10^3^ platelet/µL; the platelet concentration was always at least four times greater than the initial value, 2–5.2×10^6^ platelet/µL). In agreement with the PAW (Platelet, Activation, and White cells) classification system of PRP, we can codify our preparation as P4-x-Bβ (Platelet concentration >1,250,000 =  P4; exogenous = x, Total White Blood Cells ≤ baseline = B; Neutrophils ≤ baseline = β) [15]. This platelet enriched preparation was activated with 20 µL of 10% calcium chloride (Braun, Melsungen, Germany, 1,000 IE/mL CaCl2-2SG) at 37°C and, after jellification, immediately inserted using tweezers into the injured muscle of the same animals from which the blood had been drawn (see panel C-H in Figure S1). The wound was then sutured and washed with saline solution.

### RNA extraction and real-time PCR analysis

Total RNA was obtained from regenerated tissue at day 2 and 5 (half muscle, n = 5 in each group) using TRIZOL (Invitrogen) according to the manufacturer's procedure.

Real-time quantitative RT-PCR was performed on a 7500 Real Time PCR System (Applied Biosystems, Life Technologies). Each 20 µL reaction mixture contained 10 µL of Power SYBR Green RNA-toCt 1stepMaster mix (2×) (Life Technologies), 10p mol of specific primer sets (see Table 1), 0.16 µL RT Enzyme Mix (125×) (Life Technologies), 10–15 ng of RNA samples. The RT-PCR amplification profile was as follows: RT step at 48°C for 30 minutes, followed by enzyme activation at 95°C for 10 minutes, and then 40 cycles of denaturation at 95°C for 15 seconds and annealing/extension at 60°C for 1 minute.

**Table 1:** Rat-specific primer pair sequence for real-time PCR.

Gene	Primer pair sequence	base pair
(NCBI Reference Sequence)		
MyoD1	F: GCGACACGCGATGACTTCTAT	73
(NM_176079.1)	R: GGTCCAGGTCCTCAAAAAAGC	
Pax7	F: GCCCTCAGTGAGTTCGATTAGC	70
(NM_001191984.1)	R: TCCTTCCTCATCGTCCTCTTTC	
Myf5	F: GGCTGGTCACTGCCTCATGT	70
(NM_001106783.1)	R: CTTGCGTCGATCCATGGTAGT	
Myogenin	F: GACCCTACAGGTGCCCACAA	70
(NM_017115.2)	R: ACATATCCTCCACCGTGATGCT	
Mrf4 (myf6)	F: GCCCCTTTCCGCCTAATC	71
(NM_013172.1)	R: ACTAAGTCTCTTGCCTTTCATAAATTCTG	
VEGF-A		55
Variant 1 (NM_031836.2)	F: TACCTCCACCATGCCAAGTG	
Variant 2 (NM_001110333.1)	R: TCTGCTCCCCTTCTGTCGTG	
Variant 3 (NM_001110334.1)		
TGF-β1	F: CCACGTGGAAATCAATGGGA	91
(NM_021578.2)	R: GGCCATGAGGAGCAGGAAG	
IGF-1Eb	F: GGAGGCTGGAGATGTACTGTGCT	138
(NM_001082478)	R: TCCTTTGCAGCTTCCTTTTCTTG	
TNFα	F: AACACACGAGACGCTGAAGT	93
(NM_012675)	R: TCCAGTGAGTTCCGAAAGCC	
IL-6	F: GCAAGAGACTTCCAGCCAGT	88
(NM_012589.1)	R: AGTCTCCTCTCCGGACTTGT	
IL-1β	F: TGACTTCACCATGGAACCCG	66
(NM_031512)	R: TCCTGGGGAAGGCATTAGGA	
IL-10	F: AAGGGTTACTTGGGTTGCCA	67
(NM_012854.2)	R: GGGGCATCACTTCTACCAGG	
β-actin	F: GATCATTGCTCCTCCTGAGCG	92
(NM_031144.3)	R: TGCTGATCCACATCTGCTGGA	
GAPDH	F: ATCACTGCCACCCAGAAGAC	90
(NM_017008.4)	R: GGATGCAGGGATGATGTTCT	

All samples were run in triplicate. Values obtained for each target gene were compared with values of two internal control genes (GAPDH, and β-actin). Since the values obtained were similar, the normalization was arbitrarily done utilizing GAPDH for all target genes. A threshold cycle (C_T_) was observed in the exponential phase of amplification, and quantification of relative expression levels was performed with standard curves for target genes and the endogenous control. Geometric means were used to calculate the ΔΔC_T_ (delta-delta C_T_) values and expressed as 2^-ΔΔCT^. The value of each control sample was set at 1 and was used to calculate the fold-change of target genes.

### Analysis of microRNA expression

Quantitative analysis of miR-1, 133 and 206 was performed by RNA retro-transcription and subsequent TaqMan real-time PCR, using a 7500 Real-Time PCR System as recommended by the supplier (Applied Biosystems, Life Technologies). For each retro-transcription reaction was used 20 ng of total RNA extracted from each tissue sample.

Results were expressed as a cycle threshold (CT) value. Normalized CT values were obtained by subtracting the CT value of a small noncoding RNA control gene (*RNU6B*) from the raw CT value of each analysedmiRNA. The value of each control sample was set at 1 and used to calculate the fold-change of target genes.

### Immunofluorescent staining analysis

Muscles (n = 5 in each group) were fixed in 4% paraformaldehyde for 2–4 hours and then washed in phosphate-buffered saline. Samples were dehydrated in an ascending series of graded alcohol, cleared in xylene and embedded in paraffin. Series of transverse sections of the muscles were obtained using a microtome RM2135 (Leica Microsystems, Wetzlar, Germany).

Before staining protocol, the slides were deparaffinized and rehydrated with decreasing concentrations of alcohol. The sections were subjected to the antigen retrieval, then permeabilised with 0.2% Triton X-100 and blocked in 3% BSA for 1 hour at room temperature. Primary antiboby diluted in PBS with 1% BSA was applied on sections for 1 hour at room temperature. Secondary antibody diluted in PBS with 1% BSA was applied to the slides for 1 hour at room temperature in the dark to avoid photo-bleaching. Slides were washed in PBS and subjected to staining of nuclei with Hoechst and, then mounted. To evaluate muscle regeneration, immunohistochemistry was performed with the following antibodies: Laminin (Santa Cruz Biotechnology), MyoD1 (Santa Cruz Biotechnology) and Pax7 (Santa Cruz Biotechnology). Secondary antibodies used for each immunostaining were as follows: Alexa Fluor 488 donkey anti-goatIgG (H+L) (Molecular Probes) for Pax7 and MyoD and Alexa Fluor 546 goat anti-rabbit IgG (H+L) (Molecular Probes) for laminin. A blue fluorescent dyes as Hoechst 33258 was applied for 15 minutes for nuclear staining.

### Protein Isolation and Immunoblot Analyses

Rat's skeletal muscles of each group (n = 5; half muscle, 100–200 mg) at 2- and 5-day post-injury were lysed with the appropriate buffer plus a mixture of protease and phosphatase inhibitors (Sigma Aldrich). Homogenates were all centrifuged at 14,000 *g* for 15 min at 4°C. Equal amounts of muscle proteins (15–20 µg) were separated by SDS-PAGE and transferred onto PVDF membranes (Amersham Pharmacia Biotech). Membranes were incubated overnight with the following primary antibodies: MyoD1 (1: 2,000), SRF (1: 500), myogenin (1: 2,000), phospho-ERK1/2 (1: 1,000), Hsp27 (1: 2,000), Bax (1∶1,000) and Akt (Santa Cruz) (Santa Cruz Biotechnology); phospho-p38MAPK (1: 1,000), p38 (1: 1,000), caspase 3 (1: 1,000), phospho-NF-κB p65 (Ser536) (1: 1,000), Bcl-2 (1: 1,000), phospho-Akt (Ser473) (1: 1,000), phospho-HSP27 (Ser82) (1: 1,000) and p42 MAP Kinase (1: 1,000) (ERK2) (Cell Signaling); Hsp70/72 (1∶1,000), αB-crystallin (1: 2,000) and S59 phospho-αB-crystallin (1: 2,000) (Enzo Life Science); GAPDH (1: 3,000) (Millipore). Blots were incubated with the appropriate horseradish peroxidase-conjugated secondary antibodies (1: 15,000) (Millipore), and proteins were visualized by chemiluminescence (EuroClone). Bands were quantified by Image J software. The expression of GAPDH was used as a normalizing control. Phosphorylated isoform was normalized on the amount of its total protein.

### Statistical analysis

All values were expressed as means ± SEM. Statistical analysis was performed by a two-way ANOVA and subsequent Bonferroni's least significant difference test. *p* values less than 0.05 were considered as statistically significant. All calculations were performed using the GraphPad Prism 5 software.

## Results

### Gene expression analysis of intrinsic factors in regenerating skeletal muscle of rat treated with or without PRP

**Figure 1:**
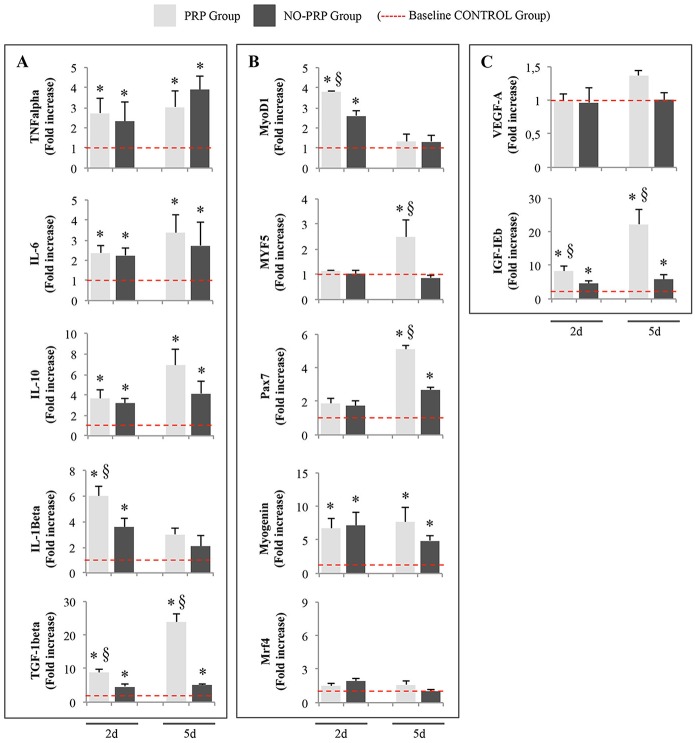
Changes in the fold increase expression levels of (A) cytokines (TNFα, IL-6, IL-10, IL-1β, and TGF-1β) (B) myogenic response factors (MyoD1, Myf5, Pax7, Myogenin and Mrf4) and (C) growth factors (VEGF-A and IGF-1Eb) mRNA in skeletal muscle treated or not with PRP during the experimental period (at day 2 and 5). The y-axis for all graphs represents the fold-difference relative to the Ctrl group. * represents significant difference between injured compared with the Ctrl group (*p*<0.05). § represents a significant difference between the PRP-injury treated group and the NO PRP-injury treated group (*p*<0.05). Values are means ± SEM (*n* = 5 rats/group at each time point). Dashed line represents the base line control group.

Real time PCR analysis revealed that mRNA transcripts for cytokines, MRFs and GFs were differently modulated from the presence of PRP (Figure 1).

As expected, at both experimental post-injury points there was an increase in the transcriptional levels of TNFα, IL-6 and IL-10 mRNA in the injured muscles when compared with control (*p*<0.05), without differences between groups treated or not with PRP (*p*>0.05) (Figure 1A).

The mRNA expression of IL-1β, was elevated at day 2 in all injured muscles (*p*<0.05), with a significantly higher transcript level in the muscle treated with PRP when compared with those left untreated (2d PRP *vs.* 2d NO-PRP 6.03 ± 0.77 *vs.* 3.6 ± 0.63, *p*<0.05). At day 5, the IL-1β mRNA level returned to approximately the same value of the Ctrl group, independently from the presence of PRP (Figure 1A).

Yet, real-time PCR analysis revealed that TGF-1β was up-regulated in injured muscles at each observation point, however, the presence of PRP makes this increase more significant even in comparison with the NO-PRP group (2d PRP *vs.* 2d NO-PRP 8.9 ± 0.8*vs.*4.5 ± 0.8, *p*<0.05; 5d PRP *vs.* 5d NO-PRP, 23.9 ± 2.2 *vs.*5.1 ± 0.3, *p*<0.05) (Figure 1A).

To further investigate myogenesis, we performed real-time PCR for several MRFs such as MyoD1, Pax7, Myf5, Myogenin and Mrf4 (Figure 1B). mRNA expression analysis showed that MyoD1, one of the earliest markers of myogenic activation and differentiation, was up-regulated at 2-day post-injury in both PRP (4-fold increase respect to the Ctrl group) and NO-PRP (3-fold increase in comparison with the Ctrl group), with a significant enhancing effect of PRP (2d PRP *vs.* 2d NO-PRP, 3.80 ± 0.01 *vs.* 2.59 ± 0.28, *p*<0.05). After 5 days, MyoD1 level returned to approximately the same level of the Ctrl group either in presence or not of PRP (5d PRP *vs.* 5d NO-PRP 1.33 ± 0.36 *vs.* 1.3 ± 0.34, *p*>0.05). Although in a different manner, even the expression of Myf5 and Pax7 were modulated by the presence of PRP. Myf5 mRNA was significantly up-regulated at day 5 only in injured muscle treated with PRP (5d PRP *vs.* NO-PRP, 2.49 ± 0.67 *vs.* 0.85 ± 0.12, *p*<0.05), while Pax7 was up-regulated at 5-day post-injury in both experimental groups, however its expression was significantly higher in PRP group when compared to the NO-PRP group (5d PRP *vs.* NO-PRP, 5.11 ± 0.24 *vs.* 2.68 ± 0.13, *p*<0.05). The expression level of Myogenin mRNA, a transcription factor important in the fusion and maturation of new muscle fibres, was increased in all injured-groups and at both sample timings, independently from PRP treatment (2d PRP 6.75 ± 1.44; 2d NO-PRP 7.18 ± 1.85; 5d PRP 7.69 ± 2.18; 5d NO-PRP 4.80 ± 0.74, *p*<0.05). No differences were detected in the expression level of Mrf4 mRNA among all groups analyzed (*p*>0.05).

The analysis of the expression of GFs involved in muscle injury such as VEGF-A and IGF-1Eb, showed that the mRNA level of VEGF-A was unchanged in all muscle-injured groups, independently from the presence of PRP (*p*>0.05) (Figure 1C). Otherwise, the level of IGF-1Eb resulted up-regulated in all experimental groups in comparison with Ctrls, with a specific enhancing effect of PRP both at day 2 (2d PRP *vs.* 2d NO-PRP, 8.34 ± 1.50 *vs.* 4.65 ± 0.71, *p*<0.05), and more specifically at day 5 (5d PRP *vs.* 5d NO-PRP, 22.20 ± 4.33 *vs.* 5.89 ± 1.46, *p*<0.05), where the IGF-1Eb mRNA expression in injured muscle tissues treated with PRP resulted 4 times higher than in not treated injured muscles (Figure 1C).

### Protein expression of MyoD1, Pax7 and Myogenin in injured muscle treated with PRP

**Figure 2:**
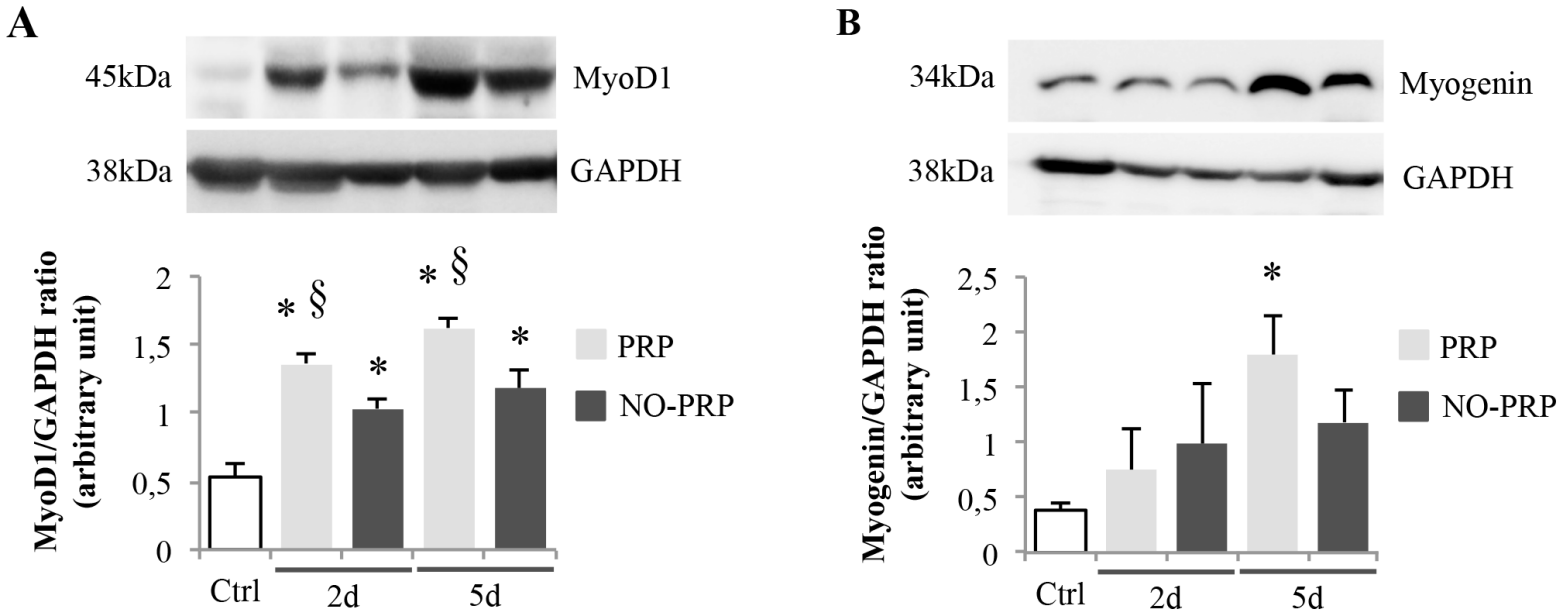
Effects of PRP on total MyoD1 (A) and Myogenin (B) protein expression in uninjured skeletal muscle of rat (Ctrl), injured-PRP treated (PRP group) or not PRP treated (NO-PRP group) at different times post-injury (at day 2 and 5). The relative protein expression was determined by the ratio of the sample value to an internal standard control (GAPDH). Values are means ± SEM (*n* = 5 rats/group at each time point). * represents a significant difference between injured groups and the Ctrl group (*p*<0.05). § represents a significant difference between PRP-injury treated groups and NO PRP-injury treated groups (*p*<0.05).

Following muscle injury, MyoD1 protein was up-regulated either in presence or in absence of PRP when compared to uninjured muscle (*p*<0.05) However, at each point investigated (at day 2 and 5), the levels of MyoD1 protein were higher in PRP with respect to NO-PRP groups (2d PRP *vs.* NO-PRP group, 1.35 ± 0.09 *vs.* 1.02 ± 0.08; 5d PRP *vs.* NO-PRP group, 1.62 ± 0.09 *vs.* 1.18 ± 0.13, *p*<0.05) (Figure 2A). A different behavior was observed when analyzing the modulation of Myogenin protein: although there was a tendency to increase at day 2 after muscle injury (Ctrl *vs.* PRP *vs.* NO-PRP group, 0.38 ± 0.07 *vs.* 0.75 ± 0.38 *vs.* 0.99 ± 0.54, *p*>0.05), a significant induction could be detected only at day 5 in presence of PRP (Ctrl *vs.* PRP group, 0.38 ± 0.07 *vs.* 1.79 ± 0.36, *p*<0.05) (Figure 2B).

**Figure 3:**
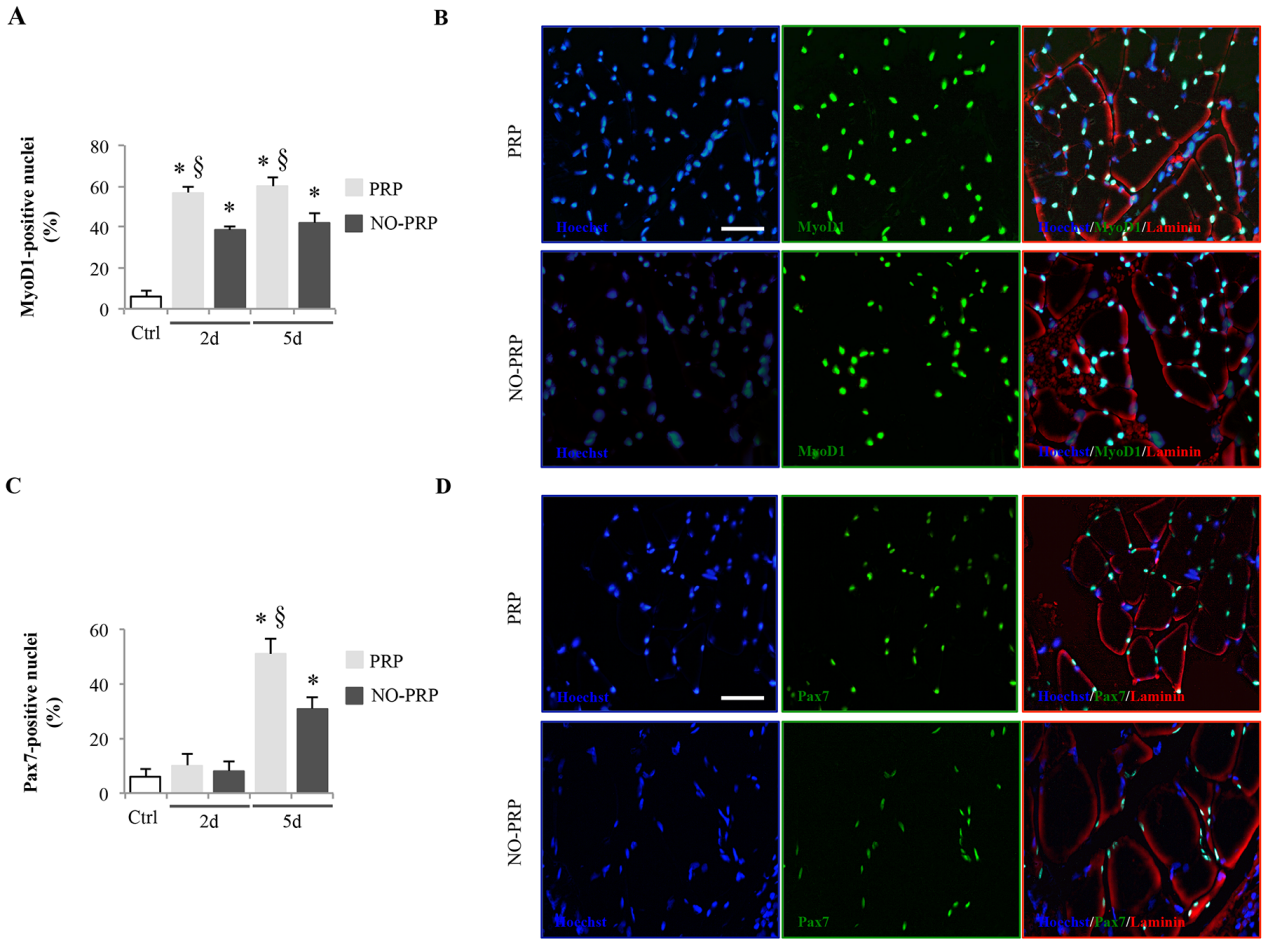
Immunohistochemical analysis of (A) MyoD1-positive or (C) Pax7-positive nuclei in skeletal muscle injury treated or not with PRP. Representative double-immunoflorescence staining of skeletal muscle for (B) MyoD1 (green) and laminin (red) or (D) Pax7 (green) and laminin (red), at day 2- and 5, respectively. Myonuclei were counterstained by blue fluorescent dyes (Hoechst). The percentage of MyoD1-positive or Pax7-positive cells was calculated as the ratio of the number of nuclei in MyoD1- or Pax7 positive cells over that of Hoechst-positive nuclei. Results were presented as means ± SEM from *n* = 5 rats/group per time point and on five sections from each animal. Scale bars  = 50 µm. * *p*<0.05 *vs*. Ctrl group. ^§^ *p*<0.05 *vs.* NO-PRP group.

The enhancing effect of PRP on MyoD1 and Pax7 was also verified through immunohistochemical analysis. Figure 3 shows the modification in the population of MyoD1- and Pax7-positive nuclei relative to the total myonuclei during the regenerating process of injured skeletal muscle at day 2 and 5, respectively. The number of MyoD1-positive nuclei was significantly increased when compared to the NO-PRP group already at 2-day post-treatment (% of MyoD1-positive nuclei: PRP *vs.* NO-PRP group, 57 ± 3 *vs.* 39 ± 2, *p*<0.05), remaining significantly more expressed even at day 5 (% of MyoD1-positive nuclei: PRP *vs.* NO-PRP group, 60 ± 4.5 *vs.* 42 ± 5, *p*<0.05)(Figure 3A-B). In accord with the qRT-PCR analysis, the effect of PRP on the number of Pax7-positive nuclei was observed only at 5-day post-injury (% of Pax7-positive nuclei: PRP *vs.* NO-PRP group, 51.0 ± 2*vs.* 31 ± 1.5, *p*<0.05) (Figure 3C-D).

### Effects of PRP on the post-injury expression pattern of muscle-specific miRNAs involved in skeletal muscle regeneration

**Figure 4:**
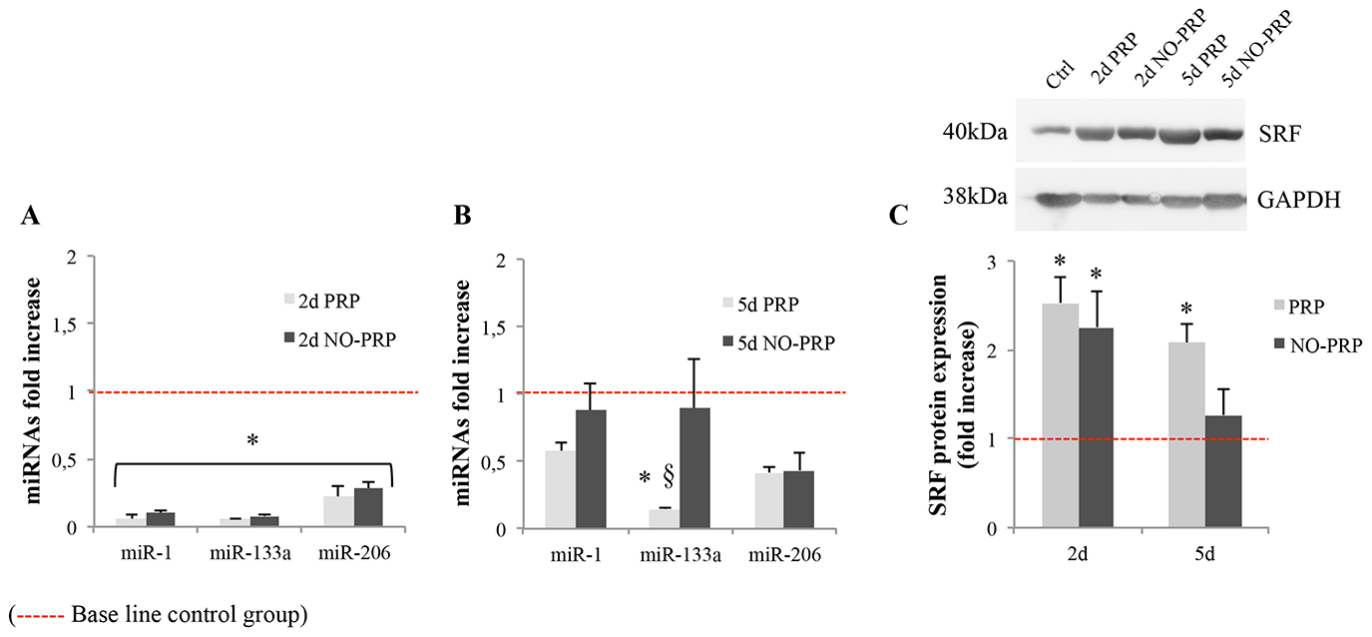
Real time-PCR analysis of miR-1, miR-133a and miR-206 expression using total RNA isolated from Ctrl-, PRP- and NO-PRP- group at 2 (A) and 5 (B) day post-injury. C) Western blot analysis of SRF protein expression in skeletal muscle at 2- and 5-day post-injury. The histograms represent fold change expression calculated as means ± SEM (*n* = 5 rats/group at each time point) respect to the Ctrl group. *p<0.05 *vs.* Ctrl group. §*p*<0.05 *vs.* 5d NO-PRP group. Dashed line represents the base line Ctrl group.

At day 2 after injury, the expression level of miR-1, miR-133a and miR-206 was significantly decreased, more than 0.5-fold with respect to the pre-injured level (*p*<0.05), independently from the presence or not of PRP (Figure 4A). At day 5, the expression level of each miRNA in the NO-PRP group was increased, returning to approximately the same level as that of pre-injury (*p*>0.05), whereas in the PRP group only the miR-1 and miR-206 levels were back to the Ctrl group magnitude (*p*>0.05). Differently, miR-133a was still significantly lower when compared to both the Ctrl and NO-PRP groups (*p*<0.05) (Figure 4B).

We then analyzed, under the same experimental condition, the protein expression of SRF, the most reliable molecular target of miR-133. Indeed, at day 2 SRF protein level was significantly increased with respect to the Ctrl group (PRP, 2.52 ± 0.29; NO-PRP, 2.25 ± 0.41, *p*<0.05), without differences between the two experimental groups (*p*>0.05) (Figure 4C). At 5-day post-injury, while in the PRP group the level of SRF was still significantly higher than in the Ctrl group, in the NO-PRP group the level of SRF protein returned to approximately the same level as in uninjured group (PRP, 2.01 ± 0.19, *p*<0.05; NO-PRP 1.26 ± 0.28, *p*>0.05) (Figure 4C).

### Analysis of p38MAPK, ERK and AKT activations in post-injury muscle tissue treated or not with PRP

**Figure 5:**
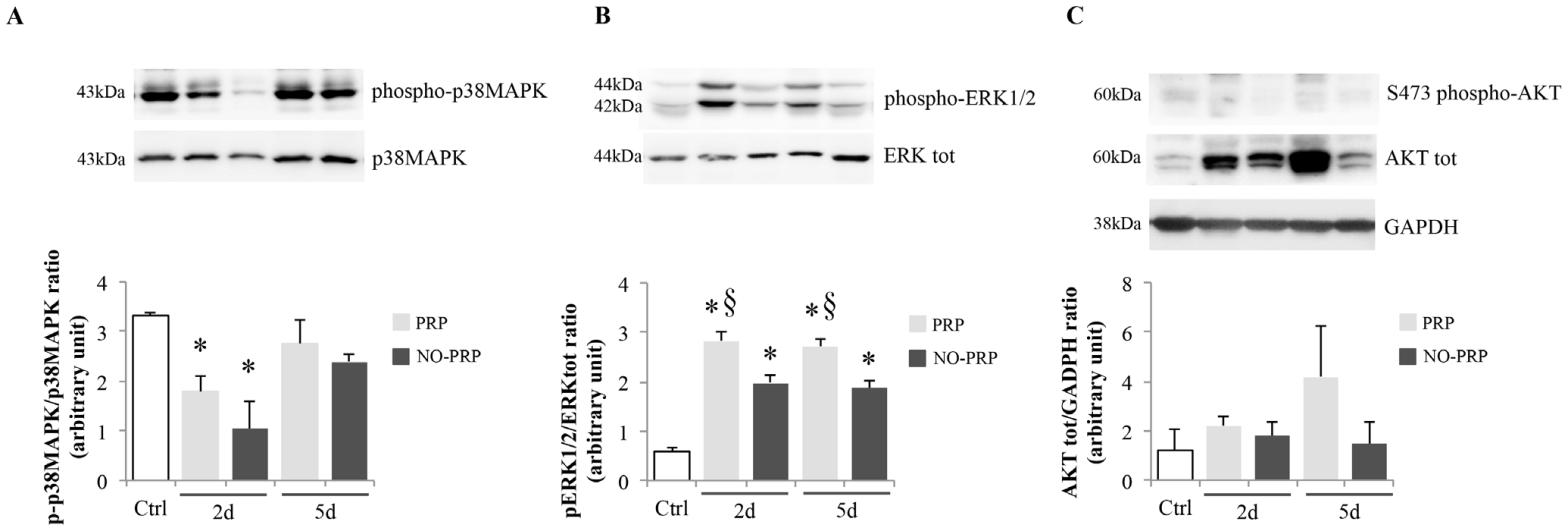
Effects of PRP on (A) p38MAPK, (B) ERK activity and (C) AKT tot at 2- and 5-day post-injury in regenerating skeletal muscle. Bar diagrams representing the densitometric intensities of p-p38MAPK, pERK1/2 and AKT tot normalized with those for p38MAPK, ERK and GAPDH content, respectively. Results were presented as means ± SEM from *n* = 5 rats/group per time point. *p<0.05 *vs.* Ctrl group. §*p*<0.05 *vs.* NO-PRP group.

The evaluation of several signaling pathways, involved at different times during muscle regeneration process, showed that only ERK activation was modulated by the presence of PRP while no effect on p38MAPK and AKT activation was observed (Figure 5). As expected, a significant decrease in p38MAPK phosphorylation at day 2 (Ctrl *vs.* 2d PRP *vs.*2d NO-PRP group, 3.32 ± 0.28 *vs.* 1.79 ± 0.09 *vs.* 1.05 ± 0.18, *p*<0.05), followed by a later recovery at day 5 (Ctrl *vs.* 5d PRP *vs.* 5d NO-PRP group, 3.32 ± 0.28 *vs.* 2.75 ± 0.44 *vs.* 2.37 ± 0.06, *p*>0.05), was observed with respect to the Ctrl group (Figure 5A). Differently, the presence of PRP induced a higher rate of ERK1/2 phosphorylation both at 2- and 5-day post-injury (2d PRP *vs.* 2d NO-PRP group, 2.82 ± 0.19 *vs.* 1.97 ± 0.17, *p*<0.05; 5d PRP *vs.* 5d NO-PRP group, 2.70 ± 0.15 *vs.* 1.87 ± 0.15, *p*<0.05) (Figure 5B). Although we identified a great individual variability in the expression of total AKT, with no statistical differences among experimental groups (*p*>0.05), we did not find any appreciable AKT phosphorylation, at any experimental time points and in the groups analyzed (Figure 5C).

### Effect of PRP treatment on the expression level of HSPs and apoptotic markers following skeletal muscle injury

**Figure 6:**
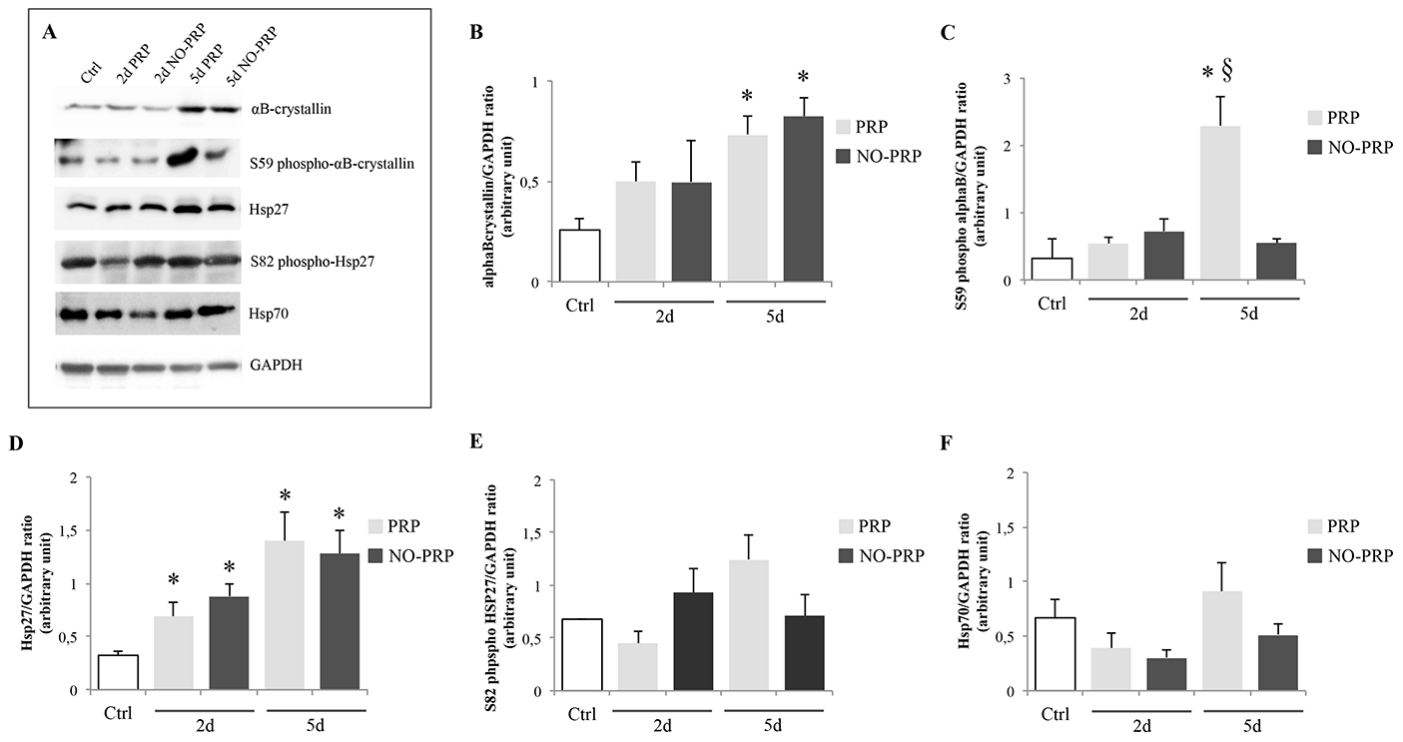
Effect of PRP treatment on several HSPs during regeneration process (2- and 5-day post-injury). (**A**) Representative immunoblot of each protein marker reported. (**B**) αB-crystallin; (**C**) S59 phospho-αB-crystallin; (**D**) Hsp27; (**E**) S82 phpspho-Hsp27; (**F**) Hsp70. Each bar represents mean value ± SEM (*n* = 5/group at each point). * *p*<0.05 *vs.* Ctrl group.§ *p*<0.05 *vs.* 5d NO-PRP group.

Changes in the mean protein expression levels of HSPs are shown in Figure 6. The level of αB-crystallin protein in muscle injured was greater than in the Ctrl group at 5-day post-injury, independently from the presence of PRP (Ctrl *vs.* 5d PRP, 0.25 ± 0.06 *vs.* 0.73 ± 0.09; Ctrl *vs.* 5d NO-PRP group, 0.25 ± 0.06 *vs.* 0.82 ± 0.09, *p*<0.05) (Figure 6B), while the effect of PRP was evident on the activation of this sHSP. Indeed, the level of phospho-αB-crystallin observed in PRP group 5 days after PRP application was 4 times greater than in NO-PRP group (Ctrl *vs.* 5d PRP group, 0.32 ± 0.28 *vs.* 2.28 ± 0.07, *p*<0.05; 5d PRP group *vs.* 5d NO-PRP group, 2.28 ± 0.07 *vs.* 0.55 ± 0.10, *p*<0.05) (Figure 6C).

**Figure 7:**
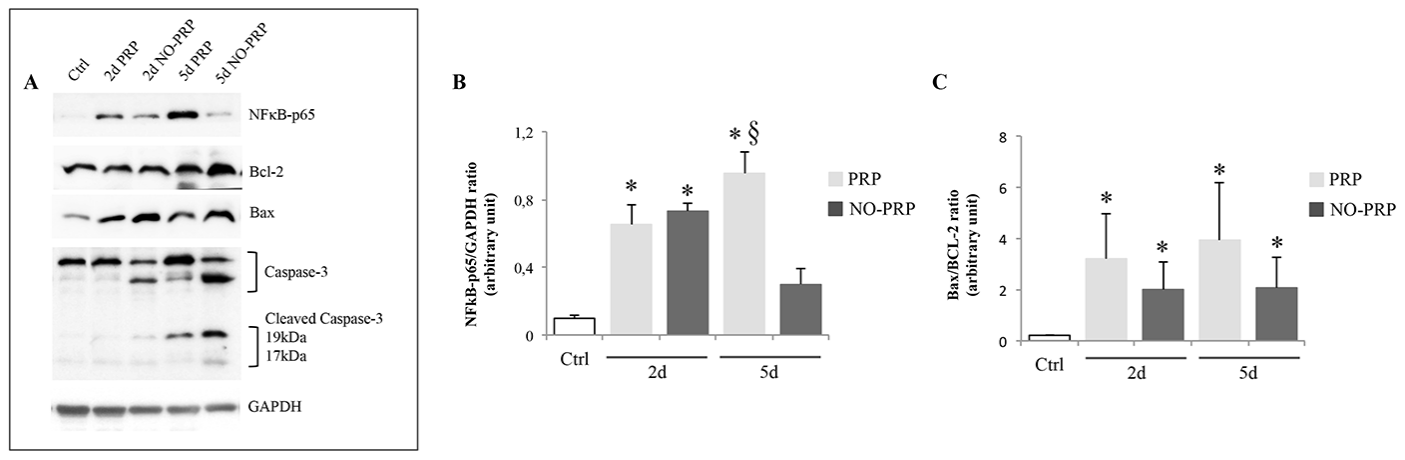
Effect of PRP treatment on several apoptotic markers during regeneration process (2- and 5-day postinjury). (**A**) Representative immunoblot of each protein marker reported; (**B**) NF-κB-p65, and (**C**) Bax/Bcl-2 ratio. Values are means ± SEM (*n*  =  5/group at each point). * *p*< 0.05 *vs.* Ctrl group.§ *p*< 0.05 *vs.* 5d NO-PRP group.

The level of Hsp27 protein in all injured groups and at both sample timings was significantly higher when compared to the uninjured group (Ctrl *vs.* 2d PRP group; 0.32 ± 0.03 *vs*. 0.69 ± 0.14, *p*<0.05; Ctrl *vs.* 2d NO-PRP group, 0.32 ± 0.03 *vs.* 0.87 ± 0.11, *p*<0.05; Ctrl *vs.* 5d PRP group, 0.32 ± 0.03 *vs.* 1.40 ± 0.28, *p*<0.05; Ctrl *vs.* 5d NO-PRP group, 0.32 ± 0.03 *vs*. 1.28 ± 0.22, *p*<0.05), independently from PRP treatment (Figure 6D). No significant differences in Hsp27 protein phosphorylation among groups were identified, even though an upward trend was observed at day 5 in presence of PRP (Ctrl *vs.* 5d PRP group, 0.68 ± 0.01 *vs.* 1.24 ± 0.23, *p* = 0.061) (Figure 7E).

There was no significant difference in the expression level of Hsp70 protein among all three groups analyzed (*p*>0.05) (Figure 6F).

Our results also identified a significant increase of NF-κB-p65 at 2-day post-injury without any effect of PRP treatment (Ctrl *vs.* 2d PRP, 0.09 ± 0.02 *vs.* 0.65 ± 0.12, *p*<0.05; Ctrl *vs.* 2d NO-PRP, 0.09 ± 0.02 *vs.* 0.73 ± 0.04, *p*<0.05). On the contrary, at 5-day post-injury, while in PRP group the level of NF-κB-p65 was still significantly higher than in the Ctrl group, in the NO-PRP group the NF-κB-p65 protein returned to approximately the same level as in the Ctrl group (5d PRP *vs.* 5d NO-PRP group, 0.95 ± 0.13 *vs.* 0.29 ± 0.09, *p*<0.05) (Figures 7A-B).

The analysis of apoptotic markers revealed that, in injured muscles, although the Bax/Bcl-2 ratio was significantly increased when compared to the Ctrl group without any modulation induced by PRP (*p*<0.05) (Figures 7A-C), the level of cleaved-caspase 3 was reduced at all time points in PRP-group (*p*<0.05) (Figure 7A).

## Discussion

The capacity of adult muscle tissue to regenerate in response to injury represents an important homeostatic process. Despite major advances in medicine, no optimal therapy has yet emerged, but the need for evidence-based strategies to regenerate or at least accelerate the regeneration of the muscle tissue still remains considerable.

To our knowledge, this is the first report describing in detail how the application of PRP to animal skeletal muscle injury can modulate, at early stages, specific molecular pathways linked to the muscle regeneration process. Indeed, we analysed multi-directionally effects of PRP on skeletal muscle regeneration, including the response of several MRFs, cytokines, GFs and micro-RNAs related to myogenesis *in vivo*. We also considered the effects of PRP in regulating stress response proteins, such as HSPs and apoptotic markers, which play a part in the tightly regulated system for the maintenance of myogenic cell homeostasis, as well as for their survival and differentiation.

Skeletal muscle regeneration consists of two overlapping stages: muscle fiber degradation, accompanied by an inflammatory response, and a reconstruction phase, which starts with the activation of muscle precursor cells, i.e. satellite cells, followed by their proliferation and differentiation of satellite cells-derived myoblasts into muscle fibers [16]. However, it must be considered that the study of muscle regeneration progress in experimental models might be influenced by the methods of injury (i.e., myotoxin, mechanical, etc.) as well as by the type of regenerating muscle under analysis [17], [18].

The early phase of muscle injury is usually accompanied by the activation of inflammatory cells residing within the muscle, which release pro- and anti-inflammatory cytokines such as TNFα, IL-6, IL-10, IL-1β and TGF-β1. These cytokines play an important role in cell proliferation, chemotaxis and cell differentiation. After skeletal muscle injury, TNFα is released not only by infiltrating macrophages, but also by injured muscle fibers [19]. Its expression remains at high levels during the repair process and returns to normal levels several days after the injury [20]. With similar time course of expression, skeletal muscle produces IL-6 in response to the injury, acting both as a factor for the proteolysis of damaged myofibers and as a signal for satellite cell proliferation [20], [21]. Although in our model we found a post-injury up-regulation of TNFα, IL-6 and IL-10, a known cytoprotective cytokine that is endogenously associated with improved muscle regeneration [22], no differences were detected with respect to the utilization of PRP. On the contrary, PRP treatment increased significantly the levels of others early pro-inflammatory cytokines, IL-1β, and of TGF-β1, a controversial secreted cytokine that, although known to be up-regulated in response to injury [23], has been related to the inhibition of myogenic differentiation and to the formation of fibrosis following muscle injury [24], [25]. Indeed, there is theoretical deleterious side effect of PRP based on the elevation of TGF-β1 levels after its injection/application into muscle with possible fibrotic healing response [26]. Therefore, since fibrotic healing following muscular injury can lead to an increased incidence of re-injury, it is important to advocate caution when considering PRP injections in muscular injury. However, it should be taken into consideration that *in vivo* studies demonstrate that TGF-β1 can inhibit, induce or have no effect on satellite cells proliferation, depending on the concomitant presence of other GFs [27], [28].

Since analysis of the effect of PRP in the late stages of muscle regeneration overcomes the aim of this study, we cannot expect whether in this experimental conditions the gel application induces a more helpful restoration of normal muscle tissue. Further studies are needed to demonstrate if our preparation of PRP induces not only a proper formation of muscle tissue but also a more rapid recovery after injury.

Following the inflammatory response, activation of normally quiescent satellite cells (SCs) is an integral part of the repair process [29]. This process is characterized by an increased transcription of the myogenic regulatory factors MyoD1, Pax7, Myf5, myogenin and Mrf4 [30].

As expected, at day 2- and/or 5 early stages of muscle regeneration, both mRNA transcripts for MyoD1, Myf5, Pax7, myogenin and an abundant number of MyoD1- and Pax7-positive cells were present in all experimental groups after muscle injury, but this response was clearly more elevated in the muscle treated with PRP, with the exception of Mrf4 mRNA and myogenin protein levels. We were not surprised to find no modulation of Mrf4 myogenic factor at the stages of regeneration selected in this study, since Mrf4 expression has been reported in the later stages of regeneration [31]. The same could also to be true for the myogenin protein level, where the lack of protein modulation may depend on the early observation window selected.

From our data, we can then hypothesize that PRP produced a major effect on muscle regeneration both through the up-regulation of Pax7 and Myf5, which leads to an increase in myogenic precursor cells and through an increase in MyoD1 expression, which is consistent with an expansion of the myogenic cell pool necessary for myofiber formation.

Collectively, these findings, together with those recently reported [6], [7], demonstrate that following a muscle injury, PRP treatment induces a marked expression of pro-inflammatory cytokines, amplifying the physiological early inflammatory response by modification of the recruitment pattern of immune cells, as well as the enhanced expression of myogenic factors, able to increase myogenesis.

Recent studies highlight the possibility that VEGF's ability to promote vascularization could increase the availability of blood vessel–associated stem cells capable of participating in muscle regeneration [32]. However, a direct improvement in muscle regeneration has not been documented. We have recently reported the absence of significant differences in both blood vessel density and diameter among PRP-treated and untreated groups [7]. Similarly, in the present study, where the same injury model was used, we did not find any specific induction of VEGF after the muscle injury, irrespective of the presence or not of PRP.

On the contrary, following imposed local damage, we found an early and huge increase of IGF-1Eb mRNA, with a clear enhanced effect induced by PRP application. It is known that IGF-1Eb acts as a survival factor by prolonging the regenerative potential of skeletal muscle not only through an increase in satellite cell activity but also establishing a balance between inflammation and connective remodeling [33]–[35]. Although defined signaling pathways, both upstream and downstream of IGF-1Eb, are still being studied, recent results suggest that IGF-1Eb is responsible for muscle progenitor cell proliferation through ERK activation but not through AKT [36], [37]. It is interesting to note that, concomitant with the up-regulation of IGF-1Eb, we found a marked phosphorylation of ERK1/2, while, despite the individual variability of AKT's expression, its phosphorylated form has never been detected.

These findings suggest that the increased expression of IGF-1Eb and the activation of ERK, leading to a higher proliferation rate of muscle progenitor cells, could represent an important molecular step for the effect of PRP during the early stages of muscle regeneration.

Growing evidences demonstrate that muscle specific miRNAs, also defined myomiRs, function as a control center in directing diverse biological processes during myogenic proliferation and differentiation [38]. Of these, the most widely studied are members of miR1, miR206 and miR-133 families [39], [40].

It is known that miR-1 promotes myogenesis by targeting histone deacetylase 4 (HDAC4), a transcriptional repressor of muscle gene expression, while miR-206 promotes muscle differentiation as well as improves skeletal muscle hypertrophy and regeneration by repressing the expression of connexin 43 (Cx43), follistatin-like 1 (Fstl1) and utrophin (Utrn) [41]–[45]. Differently, it remains controversial whether miR-133 promotes or inhibits muscle cell proliferation [40], [46], [47]. To understand if the presence of PRP was potentially able to modulate the expression of miRNAs, we analyzed the expression of miR1, miR206 and miR-133a during the early stages of skeletal muscle regeneration. Several groups have already shown that the expression levels of these miRNAs decreased subsequent to injury, thereafter their levels gradually returned to pre-injury level during the regeneration process [47]–[50]. In agreement with those data, our results showed that the level of miR-1, miR-206 and miR-133a decreased at day 2 after muscle injury in all experimental groups, returning to pre-injury levels at day 5, with the exception of miR133a, which still remained down-regulated in the presence of PRP. Among the possible targets of miR133, the most reliable is the serum response factor (SRF), which plays a critical role in muscle proliferation and differentiation depending on its association with co-factors such as myocardin, HOP, and Elk-1 [40], [47], [51]–[53].

**Figure 8:**
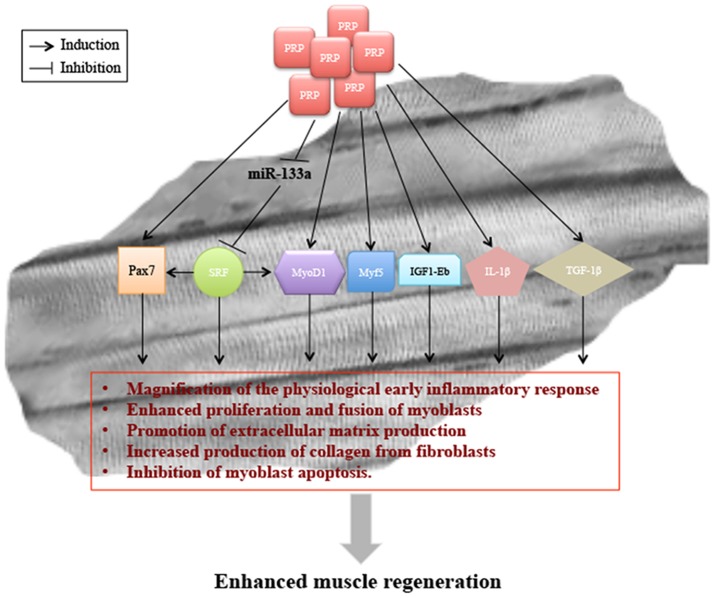
Model of PRP-mediated regulation of skeletal muscle healing. The presence of PRP modulated the expression of miR-133a and SRF protein as well as several myogenic response factors such as MyoD1, Pax7, and Myf5, the growth factor IGF-1Eb and both the cytokine IL-1β and TGF-1β. The modulation of these factors may affect important physiological processes such as the inflammatory response, myoblast proliferation and differentiation, production of extracellular matrix, and myoblast apoptosis.

The expression pattern of SRF observed in the present study seems to support the hypothesis of a specific effect of PRP on miR-133 function. Indeed, an inverse correlation between the expression levels of SRF and miR-133 was observed at least at 5-day post injury when comparing PRP versus NO-PRP or Ctrl samples. Although the differences in protein levels of SFR between PRP and NO-PRP groups were not statistically significant, the reduced content observed in NO-PRPs could still be relevant. Moreover the SRF amount in the Ctrl group, which is similar to that of NO-PRPs, was significantly lower than in the PRP group. Since is known that a high expression of miR-133 as well as a SRF reduction repress the expression of MyoD, myogenin and MyHC [40], [54], our results leave us to believe that PRP could be remarkably effective in promoting muscle regeneration by a molecular regulatory mechanism involving also miR-133-mediated up-regulation of SRF expression levels (Figure 8).

Activation of p38MAPK is a key signaling step involved in the myogenic differentiation [47], [55], [56]. Our analysis showed attenuated expression of its phosphorylated form at early stages of muscle regeneration. Besides to support the hypothesis that p38 signaling might be one of the upstream signaling regulating myomiRNAs, it is interesting to note that p38MAPK modulation was concomitant with the down-regulation of all selected myomiRNAs. As already suggested by Zhang et al (2012) [47], this phenomenon could facilitate myoblast proliferation resulting in an increased number of myoblasts ready for differentiation. Further, since it has been shown that constitutive activation of p38MAPK induce interstitial fibrosis [57], [58], its early decrease may protect skeletal muscle against fibrosis. This observation gains even more strength when considering PRP clinical application since muscle fibrosis may lead to an increased risk of re-injury.

Increased HSPs expression has been described during periods associated with enhanced protein synthesis, protein degradation and cellular reorganization, such as muscle regeneration [59]. This is especially true for two specific small heat shock proteins also defined as muscle-specific HSPs [60], Hsp27 and αB-crystallin, whose regulation is important for their role in structural cellular maintenance and molecular chaperone in myofiber stabilization [61]-[63] as well as in the resistance to apoptosis conferred during muscle differentiation [64]–[67]. It is also reported that the phosphorylation pattern of these sHSPs changes during differentiation modifying their activity, localization and interaction with other proteins and cellular functions [68]–[70].

In particular, αB-crystallin and its phosphorylated form increase NF-κB activity, whose target genes regulate biological process such as growth, differentiation and cell survival [71], [72].

In agreement with previous study [73], we found that during muscle regeneration the expression levels of both Hsp27 and αB-crystallin increased without a significant modification of Hsp70. Moreover, only in the presence of PRP we found a significant increase of phospho-αB-crystallin at 5-day post-injury.

Since a recent study showed that the up-regulation of Hsp70 is indeed important for muscle regeneration through regulating both the early inflammatory and regenerative phases following muscle injury [74], we cannot exclude that its over-expression was earlier than our observation point at day 2. However, the above cited conclusion was derived from data obtained using the cardiotoxin model of muscle injury which usually results in a high level of myofibers necrosis (greater than 90%) [75]. Therefore, the activation of all cellular pathways in cardiotoxin-injured muscles may be considerably different and/or more important than in more physiological forms of muscle injury.

**Figure 9:**
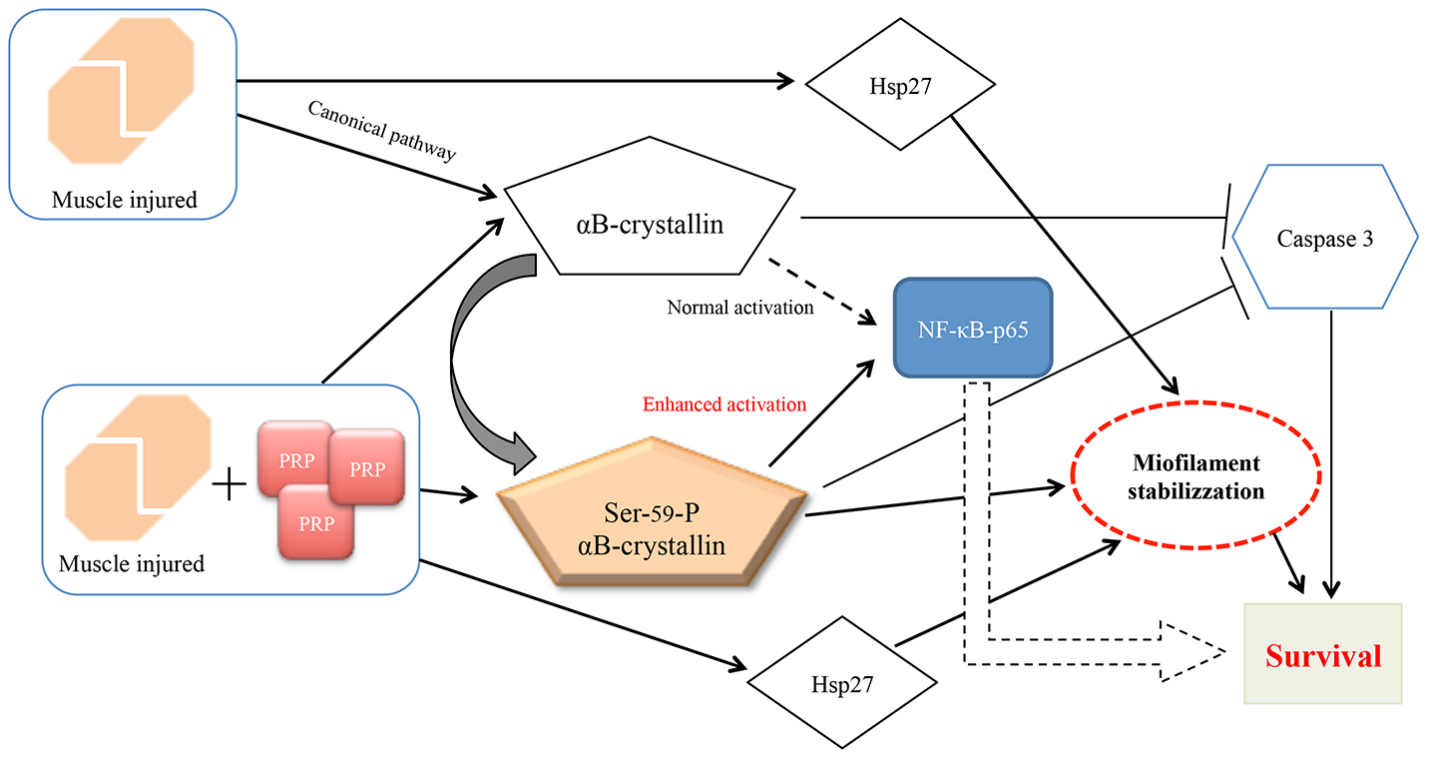
Schematic representation of the role of αB-crystallin and Hsp27 in the myofiber stabilization and in cytoprotection following skeletal muscle injury. The presence of PRP enhances phosphorylation of Ser-59 of αB-crystallin, which binds myofilaments and the inactive precursor of caspase 3, causing their stabilization and inhibition of apoptosis. Further, phospho Ser-59 αB-crystallin enhances NF-κB-p65 activation which may contribute to increased cell survival during regeneration process.

It is interesting to note that the ratio Bax/Bcl-2 was not different in presence or not of PRP, however, the expression of NF-κB p65 was higher where the lesion was treated with PRP with the concomitant reduction in the amount of cleaved caspase 3. As already hypothesized by other authors [76], [77], even under these stress conditions, we can image a similar mechanism by which Hsp27, αB-crystallin and its phosphorylated form inhibit apoptosis. Following muscle injury, serine-59 is phosphorylated causing a translocation of αB-crystallin to the myofilaments and nucleus where it binds titin, desmin, vimentin, nebulette, and inactive precursor of caspase 3, leading to the stabilization of the myofilament and to the inhibition of apoptosis [76], [77]. Although also not phosphorylated αB-crystallin is still capable to activate NF-κB, its phosphorylated form enhances even more NF-κB activity, conferring increased protection to the cells during regeneration process [77] (Figure 9).

## Conclusion

Application of PRP for musculoskeletal injuries is relatively new, and although its injections resulted in improved function, diminished pain and swelling with a shorter time of restoration and rehabilitation [14], [78]-[80], a number of important questions when considering its efficacy, safety as well as the ideal PRP formulation and frequency of injections/applications, require appropriate scientific investigation. Currently, the majority of human clinical studies show good results treating tissue pathology with one PRP injection/application [81]-[87], while in case of others a higher number was needed [5], [88]–[92].

As already hypothesized [6], [90], [93], the variability observed in PRP response profiles may be explained by the different anatomical and physiological characteristics of tissues analysed, as though the type of tissue and grade of tissue pathology heal with a different PRP request. Nevertheless, all studies above cited report an enhanced healing and functional recovery, reduced pain and increased function, no wound complications and no adverse events.

PRP has been demonstrated for over 20 years to be a safe and effective treatment option in both human and animal studies; its autologous nature eliminates any concerns of immunogenic reactions or disease transfer [94]. No studies have documented that PRP promotes hyperplasia, carcinogenesis, or tumor growth. Relative contraindications include the presence of a tumor, metastatic disease, active infections, low platelet count, pregnancy or active breastfeeding [95]. Further, although the formal etiology remains unclear, Kaux and collegues [96] reported an adverse reaction after a local injection of PRP in type 1 diabetes patient. Thus, the balance between the benefits and the risks must be carefully evaluated before using this treatment in patients with type 1 diabetes.

We must always keep in mind that PRP should not be thought as a benign “physiological” substance but rather a manipulated and “supraphysiological” product with properties potentially quite distinct from its original state.

Nevertheless, based on our observations we strongly believe that this study, together with already published results [5]–[7], [14], [97], are making significant progress towards establishing full signaling pathway induced by PRP during muscle regeneration. Indeed, the present study also demonstrated a modulation of the inflammatory response, which may explain the pain reduction usually observed after PRP administration and account for the early mobilization of treated patients [98]-[99], it showed how PRP produces a more pronounced increase of myogenic precursor cells together with an expansion of the myogenic cell pool necessary for myofiber formation. Moreover, it demonstrated that PRP is able to modulate positively even the expression of stress response proteins, directly or indirectly correlated with the regeneration process. However, this study still has several limitations. The first and most obvious is that findings from animal studies are not always applicable to humans. Second, even though the macroscopic observation allowed us to establish the timing of muscle recovery following the lesion [7], we did not analyze neither the PRP's effect on functional recovery nor the force of contraction beyond selected experimental points. Moreover, it would be interesting to perform an ultrasonography analysis in order to illustrate the enhanced regeneration in the absence of fibrosis.

Therefore, before a clinical trial can be carried out, additional studies are essential to demonstrate the efficacy and safety of PRP application as well as the possible combined/synergic effect of PRP with specific training protocol, such as the eccentric physical therapy [100]. If elucidated, this concept of mechanical stimulation after PRP treatment could be beneficial to human soft tissue and other pathologies that are sensitive to variation in local tissue strength.

## Supporting Information

Figure S1 **Surgical and delivery procedure of platelet gel in injured muscle.** **A**) Withdrawal of intracardiac blood; **B**) Transfer blood into vacutainer tubes with sodium citrate for centrifugation steps; **C**) Multiwell with platelet gel; **D**) Incision upper limb; **E**) Identification upper limb flexor muscle; **F**) Platelet gel; **G**) Delivery of the platelet gel; **H**) Transplant of platelet gel in injury flexor muscle.
(TIF)
