# Supplementary figures and images for: Platelet-Rich Plasma and Skeletal Muscle Healing: A Molecular Analysis of the Early Phases of the Regeneration Process in an Experimental Animal Model
Source: PLoS One. 2014 Jul 23;9(7):e102993. doi: 10.1371/journal.pone.0102993 (PMC4108405; doi:10.1371/journal.pone.0102993)

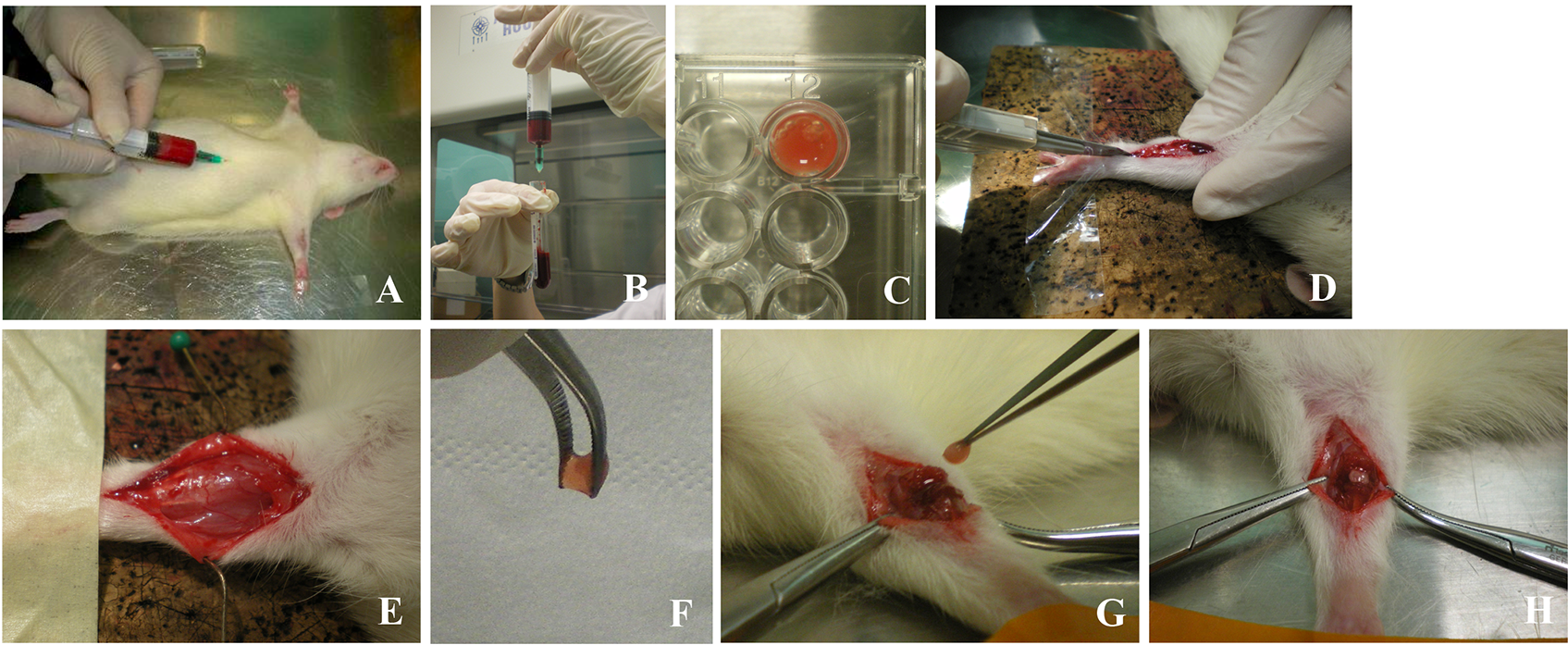

Supplement: Figure S1 — Surgical and delivery procedure of platelet gel in injured muscle. A) Withdrawal of intracardiac blood; B) Transfer blood into vacutainer tubes with sodium citrate for centrifugation steps; C) Multiwell with platelet gel; D) Incision upper limb; E) Identification upper limb flexor muscle; F) Platelet gel; G) Delivery of the platelet gel; H) Transplant of platelet gel in injury flexor muscle. (TIF) [file pone.0102993.s001.tif]
